# Editorial: Insights Into Biomarkers, Cytokines, and Chemokines in Skin Cancer

**DOI:** 10.3389/fmed.2019.00199

**Published:** 2019-09-25

**Authors:** Lucy W. Barrett, Elin S. Gray, James W. Wells, Jason Waithman

**Affiliations:** ^1^Telethon Kids Institute, Perth Children's Hospital, University of Western Australia, Nedlands, WA, Australia; ^2^Institute for Respiratory Health, Sir Charles Gairdner Hospital, Nedlands, WA, Australia; ^3^School of Medical and Health Sciences, Edith Cowan University, Joondalup, WA, Australia; ^4^The University of Queensland Diamantina Institute, The University of Queensland, Woolloongabba, QLD, Australia; ^5^Dermatology Research Centre, The University of Queensland, Woolloongabba, QLD, Australia

**Keywords:** skin cancer, immunology, biomarkers, chemokines, cytokines, immunotherapy, skin, lymphocyte trafficking

As our understanding of immune system involvement in skin cancer has increased, so has the development of immunotherapies to target aggressive, un-resectable, or metastatic skin cancers, resulting in significant improvements in survival for previously untreatable patients. However, the high rate of non-responders and the development of resistance to immunotherapy over time highlights the need to improve the efficacy of these treatments. The skin microenvironment is unlike other non-lymphoid organs in so far as lymphocytes constantly traffic to and from the skin and immune surveillance occurs even in the absence of infection (Lafouresse and Groom). Thus, the immune system plays a critical role in the maintenance of a healthy skin microenvironment, and disruption of immune surveillance mechanisms is thought to contribute to multiple skin neoplasia's, including cancer. Our current Research Topic highlights the complexity of the relationship between the skin, immune system and skin cancer, and underlines the need to characterize the specific molecular mechanisms involved in cell trafficking in a context-specific manner. Here we provide a brief overview of the five review articles and two original research papers that contributed to our recent Research Topic, focusing on general ideas that have emerged and implications for future research in the field.

A major theme running through this topic is the dissection of molecular mechanisms involved in lymphocyte trafficking into the skin and tumors, with a particular focus on the specific chemokines involved (Lafouresse and Groom; Yam and Chtanova; Kuo et al.). Trafficking of immune cells into the skin is a complex and highly regulated process as each cell type responds to a specific set of signals, and chemokines and their receptors can be variably expressed by both normal skin cells and malignant cells. In the review by Kuo et al., the role of the chemokine receptor CXCR3 and associated ligands in interferon-induced inflammatory responses and skin cancer is examined in detail, and describes how CXCR3 expression on immune cells often promotes anti-tumor responses, while conversely, expression on tumor cells can lead to metastasis. Evidence for the opposing roles of immune cells in cancer progression and metastasis is further described in the mini review by Yam and Chtanova, in which the roles of other chemokine receptors and ligands in the trafficking of dendritic cells, macrophages, neutrophils and natural killer cells into tumors is examined. In addition to the movement of lymphocytes, Ju et al. further address the pathways involved in metastasic spread, with chemokines and their receptors remaining a key focus. Importantly, the authors discuss the concept that the mechanisms behind metastatic spread may also enhance tumor cell survival, making these pathways attractive targets for future therapies.

Tumors that are rich in cytotoxic T cells tend to be associated with a better prognosis and response to immunotherapy (Lafouresse and Groom; Yam and Chtanova). However, the role of innate immune cells including dendritic cells, macrophages, neutrophils, and natural killer cells in tumors is more complex, as they can mediate anti-tumor responses through the recruitment of cytotoxic T cells, or alternatively may inhibit anti-tumor responses through expression of suppressive factors, resulting in enhanced tumor growth (Yam and Chtanova). Even when T cells are effectively recruited to tumors, they may be inhibited by exhaustion or infiltration of suppressive T regulatory cells (Lafouresse and Groom). A greater understanding of the interplay between innate and adaptive lymphocytes, and how this ultimately influences cancer growth, is needed to promote greater response rates to immunotherapy, which is the second major theme of our Research Topic. The articles presented emphasize the urgent need to understand the specific mechanisms behind non-response or developed resistance to emerging treatments.

To improve responses and treatment regimes, biomarkers and new predictors for treatment response are urgently needed (Bridge et al.). In their review, Bridge et al. examined current research into checkpoint inhibitor therapy for various skin cancers and the advances that have been made in identifying biomarkers for non-response, tolerance, and toxicity. The authors discuss the current state of immunotherapy for metastatic skin cancers, the limitations of the current biomarkers, and address important questions pertaining to the general unpredictability of the responses to checkpoint inhibitor (CPI) therapies, such as: Can CPIs be re-administered in the case of relapse? Do all CPI treatments work through the same pathways, meaning patients who are resistant to one would be resistant to others? And when should treatment be stopped? These questions reflect some of the current priorities for improving immunotherapy outcomes.

T cell exhaustion, whereby T cells progressively lose their effector function, presents a major challenge to effective immunotherapies. An original study by Graves et al. analyzed the expression of inhibitory markers on the surface of T cells from 16 patients treated with Pembrolizumab, an anti-PD-1 therapy for metastatic melanoma. They identified upregulation of PD-1 and TIM-3 on CD8 T cells as potential biomarkers to identify non-responders to Pembrolizumab therapy. The second original research paper presented as part of this topic also details a search for biomarkers, this time for squamous cell carcinoma (SCC). Crawford et al. identified two anthrax toxin receptors, Tumor Endothelial Marker 8 (TEM8) and Capillary Morphogenesis Gene 2 (CMG2), that are upregulated on SCC cells. Protective antigen (PA), a protein from *Bacillus anthracis* that has been shown to bind to these receptors, was fluorescently labeled and intravenously infused in a mouse model of SCC. The authors showed that they were able to detect tumor-specific uptake of PA through binding with TEM8. This research identifying TEM8 as a potential biomarker for SCC has important implications for treatment, as it could facilitate early, accurate detection of skin malignancy and enhance surgical precision of tumor removal.

The studies in this issue illustrate the complexities and challenges researchers face in designing effective and long-lasting immunotherapies for skin cancer. Through detailed studies, such as those presented here, it may well become possible to harness the immune system to destroy skin cancer and prevent its recurrence. Characterization of immune cell movements and functions within the skin and in response to immunotherapies are a priority in order to provide the best possible treatment for patients in the future.

## Author Contributions

LB prepared the initial manuscript. EG, JWW, and JW all provided feedback and edited the final manuscript.

### Conflict of Interest Statement

The authors declare that the research was conducted in the absence of any commercial or financial relationships that could be construed as a potential conflict of interest.

